# Herpes simplex virus 1 infection dampens the immediate early antiviral innate immunity signaling from peroxisomes by tegument protein VP16

**DOI:** 10.1186/s12985-017-0709-5

**Published:** 2017-02-21

**Authors:** Chunfu Zheng, Chenhe Su

**Affiliations:** 1 Institutes of Biology and Medical Sciences, Soochow University, Suzhou, 215123 China; 2 Department of Microbiology, Immunology and Infectious Diseases, University of Calgary, Calgary, AB T2N 4N1 Canada

**Keywords:** HSV-1, VP16, MAVS-Pex, Immune evasion

## Abstract

**Background:**

Herpes simplex virus 1 (HSV-1) is an archetypal member of the alphaherpesvirus subfamily with a large genome encoding over 80 proteins, many of which play a critical role in virus-host interactions and immune modulation. Upon viral infections, the host cells activate innate immune responses to restrict their replications. Peroxisomes, which have long been defined to regulate metabolic activities, are reported to be important signaling platforms for antiviral innate immunity. It has been verified that signaling from peroxisomal MAVS (MAVS-Pex) triggers a rapid interferon (IFN) independent IFN-stimulated genes (ISGs) production against invading pathogens. However, little is known about the interaction between DNA viruses such as HSV-1 and the MAVS-Pex mediated signaling.

**Results:**

HSV-1 could activate the MAVS-Pex signaling pathway at a low multiplicity of infection (MOI), while infection at a high MOI dampens MAVS-Pex induced immediately early ISGs production. A high-throughput screen assay reveals that HSV-1 tegument protein VP16 inhibits the immediate early ISGs expression downstream of MAVS-Pex signaling. Moreover, the expression of ISGs was recovered when VP16 was knockdown with its specific short hairpin RNA.

**Conclusion:**

HSV-1 blocks MAVS-Pex mediated early ISGs production through VP16 to dampen the immediate early antiviral innate immunity signaling from peroxisomes.

## Background

The host innate immune system plays an important role in detecting the invading pathogens. Conserved pathogen-associated molecular patterns from viruses are initially recognized by multiple immune sensors that are referred to as pattern recognition receptors (PRRs) [1–3]. Besides Toll-like receptors in the cellular membrane or endosome and Nod-like receptors in the cytoplasm, the retinoic acid-inducible gene I (RIG-I)-like receptors (RLRs) such as RIG-I and melanoma differentiation-associated gene 5 (MDA-5) are able to detect 5′-triphosphate-containing short double-stranded RNA (dsRNA) and long dsRNA respectively and activate the expression of type I interferons (IFNs) and IFN-stimulated genes (ISGs) [4–8]. RLRs can detect both viral RNA and RNA polymerase III-mediated transcription of microbial DNA [8–10]. Additionally, MDA-5 was reported to be a primary mediator of HSV recognition using small interfering RNA knockdown in HSV-infected macrophages [11]. Upon recognition of viral RNA, RIG-I and MDA-5 interact with the mitochondrial antiviral signaling protein (MAVS, also known as IPS-1, VISA, and CARDIF), which then leads to the production of IFNs and ISGs [12, 13].

As the adaptor protein downstream of the RLR-dependent signaling pathway, MAVS was reported to locate on mitochondria, peroxisomes and mitochondria-associated membranes of the endoplasmic reticulum [14–16]. Peroxisomes have been established to be metabolic regulation organelles for a long time, which control the metabolism of lipids and reactive oxygen species [17–19]. Surprisingly, these organelles were also revealed to be important signaling platforms for antiviral innate immunity. Upon viral infection, peroxisomal MAVS (MAVS-Pex) triggers an immediate early induction of ISGs, which is type I IFN-independent, whereas mitochondrial MAVS shows a delayed and sustained antiviral effect based on the induction of type I IFNs and ISGs [14]. In addition, MAVS-Pex is also identified to activate type III IFN expression [20].

Herpes Simplex Virus 1 (HSV-1) is an archetypal member of the alphaherpesvirinae subfamily, which encodes over 80 proteins. VP16, a 65-kDa tegument protein of HSV-1, was reported to have various functions, including transcriptional activation of viral immediate early genes, downregulation of the virion host shutoff protein and participation in viral egress downstream of the initial envelopment [21–24]. Furthermore, our previous study has demonstrated that VP16 also downregulates the production of IFN-β in RLR signaling pathway [25].

A variety of ISGs function as antiviral effectors. Viperin (also known as cig5 or RASD2), a highly conserved and well-characterized ISG protein, limits the replication of many DNA and RNA viruses [16, 26–37]. To survive within the infected host, HSV-1 has evolved multiple strategies to counteract host antiviral innate immune responses through its numerous proteins [25, 38–44]. In this study, we demonstrated for the first time that HSV-1 tegument protein VP16 dampened the MAVS-Pex signaling and the expression of the immediate early ISGs, such as viperin.

## Results

### HSV-1 infection triggers a MAVS-Pex-dependent early induction of viperin

**Fig. 1:**
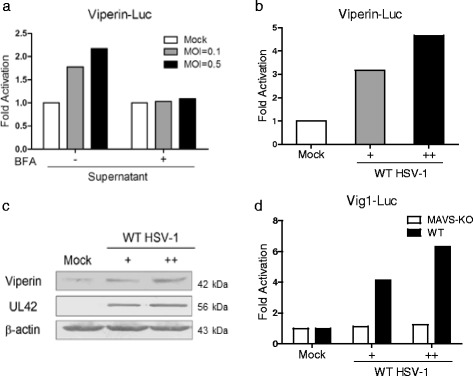
HSV-1 infection triggers a MAVS-Pex-dependent early induction of viperin. **a** HEK293 cells were pretreated with or without 50 ng/ml BFA and infected with or without WT HSV-1 at an MOI of 0.1 or 0.5. At 2 h post infection, the supernatants were discarded and replaced with fresh media containing BFA for 18 h. Then, the supernatants were collected and added to Viperin-Luc and pRL-TK transfected cells for DLR analysis. (**b** and **c**) HEK293 cells were pretreated with 50 ng/ml BFA and infected with or without WT HSV-1 at an MOI of 0.1 (+) or 0.5 (++). At 2 h post infection, the supernatants were discarded and replaced with fresh media containing BFA. At 18 h post infection, cells were harvested and subjected to RT-PCR (**b**) and WB analysis with anti-viperin antibody (**c**). **d** MAVS-KO and WT MEF cells were pretreated with 100 ng/ml BFA and infected with or without WT HSV-1 at an MOI of 0.1 (+) or 0.5 (++). At 2 h post infection, the supernatants were discarded and replaced with fresh media and the cells were transfected with Vig1-Luc and pRL-TK for 18 h in the presence of BFA and subjected to DLR analysis. The data represent results from one of the triplicate experiments

Previous study demonstrated that MAVS-Pex signaling could be activated by RNA viruses, such as reovirus and influenza virus [14]. However, whether DNA viruses such as HSV-1 could activate the MAVS-Pex signaling has not been demonstrated. Firstly, we investigated whether HSV-1 could induce the activation of the immediate early ISG gene viperin downstream of MAVS-Pex. As mentioned above, activation of mitochondrial MAVS plays an important role on the production of type I IFN, while activation of peroxisomal MAVS could trigger the production of type III IFN, both of which induce the expression of ISGs. Therefore, Brefeldin A (BFA) was applied in our experiments to disrupt type I and III IFNs secretion, thus excluding the viperin production from type I and III IFNs [14]. As shown in Fig. 1a, supernatants from wild-type (WT) HSV-1 infected cells could trigger the activation of viperin promoter, while pretreatment with BFA blocked viperin promoter activation from IFNs. HEK293 cells were pretreated with BFA and then infected with HSV-1. At 18 h post infection, cells were harvested and subjected to quantitative RT-PCR (qRT-PCR) analysis to determine viperin mRNA level. Our results showed that HSV-1 infection could successfully enhance viperin mRNA level independent of type I and III IFNs (Fig. 1b). In addition, Western blot (WB) assays were performed and similar results were obtained (Fig. 1c). To further demonstrate that the immediate early viperin induction triggered by HSV-1 infection is mediated by MAVS-Pex, we performed the dual-luciferase reporter (DLR) experiment based on mouse viperin luciferase reporter Vig1-Luc in MAVS-KO and WT MEF cells. The results showed that HSV-1 infection triggered the induction of viperin in BFA-treated WT MEF cells, but not in MAVS-KO MEF cells, which indicated that the immediate early viperin expression induced by HSV-1 infection depended on MAVS-Pex (Fig. 1d). Taken together, these results demonstrated that HSV-1 could trigger the MAVS-Pex-dependent immediate early induction of viperin.

### HSV-1 infection at a high multiplicity of infection (MOI) dampens the immediate early induction of viperin triggered by MAVS-Pex

**Fig. 2:**
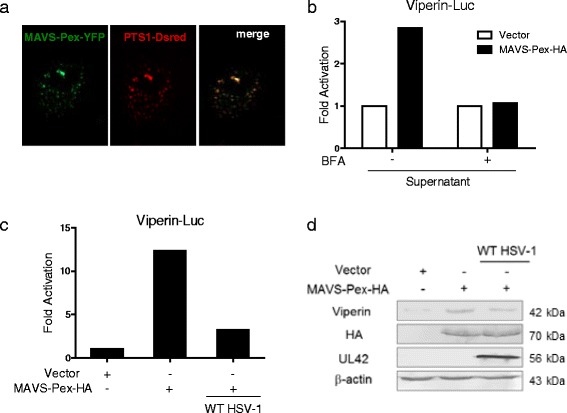
HSV-1 infection at a high MOI dampens the immediate early induction of viperin triggered by MAVS-Pex. **a** MEF cells were transfected with MAVS-Pex-YFP, along with PTS1-DsRed plasmid for 36 h. The images were obtained by fluorescence microscopy using a 20× objective. **b** HEK293 cells were pretreated with or without 50 ng/ml BFA and transfected with empty vector or MAVS-Pex-HA plasmid for 18 h. Then, the supernatants were collected and added to Viperin-Luc and pRL-TK transfected cells for DLR analysis. (**c** and **d**) HEK293 cells were pretreated with 50 ng/ml BFA and infected with or without WT HSV-1 at an MOI of 5. At 2 h post infection, the cells were transfected with the indicated plasmids in the presence of BFA. Eighteen hours post transfection, cells were harvested and subjected to RT-PCR (**c**) and WB (**d**) analysis. The data represent results from one of the triplicate experiments

As HSV-1 has evolved multiple strategies to counteract host innate immune system, we further determine the role of HSV-1 infection on the early antiviral signals triggered by MAVS-Pex. Firstly, a YFP-tagged MAVS-Pex plasmid (MAVS-Pex-YFP) was transfected into MEF cells to examine its subcellular localization, where the peroxisomes were marked by a DsRed allele containing a type 1 peroxisomal targeting signal (PTS1). As shown in Fig. 2a, MAVS-Pex-YFP is exclusively located on peroxisomes. Meanwhile, we confirmed that pretreatment with BFA was capable of disrupting IFN secretion induced by MAVS-Pex, thus excluding the viperin production from IFN (Fig. 2b). We next examined whether HSV-1 infection could affect MAVS-Pex-induced immediate early induction of viperin. HEK293 cells were pretreated with BFA and infected with WT HSV-1 at a high MOI of 5 prior to transfection of the indicated plasmids. Cells were subjected to qRT-PCR analysis 18 h post transfection. Our results showed that HSV-1 successfully blocked activation of viperin mRNA induced by MAVS-Pex-HA (Fig. 2c). Additionally, WB assays were performed to verify the result (Fig. 2d). In conclusion, the immediate early induction of viperin triggered by MAVS-Pex could be dampened by HSV-1 infection at a high MOI.

### VP16 inhibits the early induction of viperin from MAVS-Pex

**Fig. 3:**
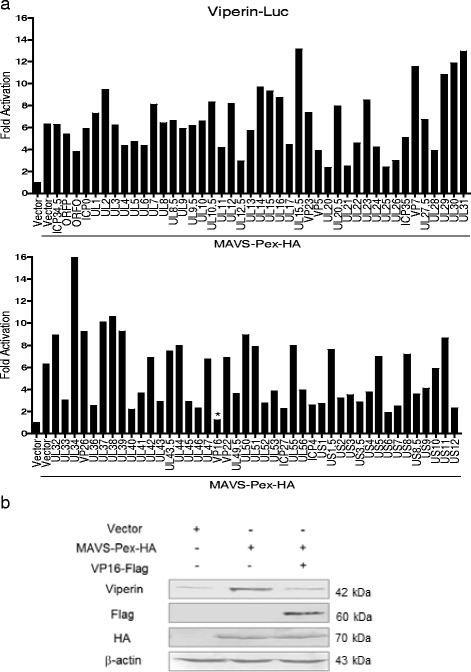
VP16 inhibits the early induction of viperin from MAVS-Pex. **a** HEK293 cells were pretreated with 50 ng/ml BFA and transfected with empty vector, MAVS-Pex-HA and individual HSV-1 protein expression plasmid in the presence of BFA. At 18 h post transfection, cells were harvested and subjected to qRT-PCR analysis. **b** HEK293 cells were pretreated with 50 ng/ml BFA and transfected with MAVS-Pex-HA and VP16-Flag plasmids as indicated in the presence of BFA. At 18 h post transfection, cells were harvested and subjected to WB analysis with anti-viperin antibody. The data represent results from one of the triplicate experiments

The data above led us to hypothesize that at least one of the HSV-1 proteins was involved in inhibiting MAVS-Pex mediated early induction of viperin. A high-throughput qRT-PCR screen assay was performed. HEK293 cells were pretreated with BFA and co-transfected with the individual HSV-1 protein expression plasmid [45]. As a result, the tegument protein VP16 was found to most effectively inhibit MAVS-Pex induced viperin mRNA expression (Fig. 3a). To further confirm the result, HEK293 cells pretreated with BFA were transfected with empty vector, MAVS-Pex-HA plasmid, or Flag-tagged VP16 plasmid together with the MAVS-Pex-HA plasmid as indicated. At 18 h post transfection, cells were harvested and subjected to WB analysis. As a result, MAVS-Pex-induced expression of viperin was strongly inhibited by VP16 (Fig. 3b). Collectively, these results demonstrated that VP16 could inhibit the immediate early induction of viperin from MAVS-Pex.

### Knockdown of VP16 restores the immediate early innate antiviral signaling triggered by MAVS-Pex or HSV-1 infection

**Fig. 4:**
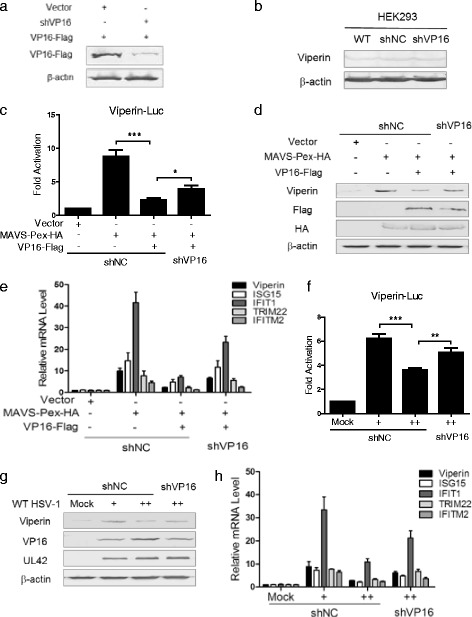
Knockdown of VP16 restores the immediate early innate antiviral signaling triggered by MAVS-Pex or HSV-1 infection. **a** HEK293T cells were transfected with VP16-Flag, shNC or shRNA-VP16 as indicated for 36 h and subjected to WB analysis with anti-Flag antibody. **b** WT HEK293, shNC and shVP16-expressing HEK293 cells were subjected to WB analysis with anti-viperin antibody. (**c**, **d** and **e**) ShNC and shVP16-expressing HEK293 cells were pretreated with 50 ng/ml BFA and transfected with the indicated plasmids for 18 h in the presence of BFA, and subjected to RT-PCR (**c**), WB (**d**) and qRT-PCR (**e**) analysis. (**f**, **g** and **h**) HEK293 cells were pretreated with 50 ng/ml BFA and infected with or without WT HSV-1 at an MOI of 0.5 (+) or 1 (++). At 2 h post infection, cells were transfected with shNC or shVP16 plasmids as indicated in the presence of BFA. At 18 h post transfection, cells were harvested and subjected to RT-PCR (**f**), WB (**g**) and qRT-PCR (**h**) analysis. The data represent results from one of the triplicate experiments. Statistical analysis was performed using Student’s *T*-test with the GraphPad Prism 5.0 software. (* 0.01 < *P*<0.05; ** 0.001 < *P*<0.01; *** 0.0001 < *P*<0.001)

We next attempted to evaluate whether the loss of HSV-1 VP16 expression would affect its ability to inhibit MAVS-Pex mediated early induction of viperin. However, UL48, which encodes the VP16 protein, is an essential gene for HSV-1 and the deficiency of it was reported to display a lethal impact on the replication and infection of HSV-1 [22]. Therefore, a short hairpin RNA (shRNA) specific for VP16 (shVP16) or a scrambled shRNA as control (shNC) were constructed. Firstly, we evaluated the knockdown effect of shVP16 in HEK293T cells (Fig. 4a). Then, HEK293 cells were transfected with shVP16 or shNC to screen for stable transfection cell lines. To rule out the possibility that the stable transfection of shVP16 alone altered viperin expression, we tested the endogenous expression of viperin in HEK293, HEK293-shNC and HEK293 shVP16 cells, and the WB result showed that the endogenous expression level of viperin in HEK293-shVP16 cells was similar to that in HEK293 and HEK293-shNC cells (Fig. 4b). Next, as shown in Fig. 4c, the induction of viperin mRNA was decreased in HEK293 shNC cells co-transfected with VP16-Flag and MAVS-Pex-HA, while in HEK293 shVP16 cells the induction of viperin mRNA was recovered. The WB assays were also performed and similar results were obtained (Fig. 4d). Furthermore, we also performed qRT-PCR analysis to explore the effect of VP16 on other immediate early ISGs mediated by MAVS-Pex. As shown in Fig. 4e, MAVS-Pex could induce other immediate early ISGs while VP16 inhibited all these ISGs (Fig. 4e). On the other hand, we investigated whether knockdown of VP16 would affect the induction of the immediate early ISGs during HSV-1 infection. HEK293 cells were pretreated with BFA and then infected with or without WT HSV-1 at an MOI of 0.5 or 1. At 18 h post infection, cells were harvested and subjected to qRT-PCR analysis. The result showed that knockdown of VP16 restored the expression of viperin dampened by HSV-1 infection at the MOI of 1 (Fig. 4f). Meanwhile, similar result was obtained in WB analysis (Fig. 4g). Moreover, as shown in Fig. 4h, HSV-1 activated other immediate early ISGs downstream of MAVS-Pex at an MOI of 0.5 and dampened all these ISGs by VP16 at the MOI of 1. In conclusion, knockdown of VP16 restored the induction of immediate early ISGs triggered by MAVS-Pex or HSV-1 infection. Therefore, during HSV-1 early infection, VP16, as an abundant tegument protein, was shown to play a major role in dampening the immediate early antiviral signaling from peroxisomes and contributing to the early-stage survival during HSV-1 infection.

## Discussion

As the first line of defense against infectious threats, the innate immune system represents a conserved role in response to virus invasion and achieves their detections through PRRs. In RLRs signaling pathway, MAVS was originally reported to reside on mitochondria and bound to RLRs to initiate the downstream activation of NF-κB and interferon regulatory factors (IRFs) [16], which then translocated to the nucleus and activated the transcription of IFNs and ISGs. Nevertheless, MAVS was also reported to reside on peroxisomes, where it could trigger a rapid, IFN-independent ISG response, completely different from that of the mitochondrial MAVS [14].

As a double-strand DNA virus, HSV-1 evolved multiple mechanisms to evade the host innate immunity and establish its infection [46]. The virion host shutoff protein blocks cellular antiviral proteins, like tetherin, viperin, and zinc finger antiviral protein, by targeting their mRNA for degradation [47–49]. Us11, an RNA binding tegument protein, interferes with the interaction between MAVS and RIG-I or MDA-5, thus dampens IRF3 activation and IFN production [38]. UL36, a ubiquitin-specific protease, inhibits IFN production by deubiquitinating TRAF3 to prevent recruitment of TBK1 [40]. Us3, which hyperphosphorylates IRF3 and p65, blocks their nuclear translocation and thus down regulates IFN-β production [41]. ICP0, an E3 ubiquitin ligase, disrupts NF-κB activation by abrogating the nuclear translocation of p65 and degrading p50 through ubiquitin-proteasome pathway [42]. ICP34.5, best known for its ability to inhibit the IFN-inducible kinase PKR, also dampens IFN production by binding and sequestering TBK-1, which phosphorylates IRF3 [50]. VP24, a serine protease of HSV-1, blocks DNA sensing signal pathway via abrogating the interaction between TBK1 and IRF3 [51].

Although there are plenty of HSV-1 proteins which block the IFN-β signaling pathway, the role of HSV-1 on the MAVS-Pex signaling has not been reported. Here we demonstrate for the first time that HSV-1 is able to activate the early antiviral signaling from peroxisomes. VP16, which is encoded by UL48 gene, has various functions in viral growth and infection. Our previous study showed that VP16 could abrogate the production of IFN-β, which presented a critical role of VP16 in mitochondrial MAVS signaling [25]. In this present study, we demonstrated another important effect of VP16 to abrogate the early expression of ISGs in the IFN-independent MAVS-Pex signaling pathway, which was characterized by viperin. As an abundant tegument protein, VP16 was released into host cells upon infection, dampened the immediate early antiviral immunity from peroxisomes in host cells and facilitated the proliferation of HSV-1. In addition, we found that VP16 could also inhibit the activation of viperin promoter induced by IRF3 and IRF1, two central regulators of IFN independent ISG expression which act downstream of MAVS-Pex (data not shown). And previous studies in our lab have reported that VP16 interacted with the CREB binding protein (CBP) coactivator and efficiently inhibited the formation of the transcriptional complexes IRF-3-CBP [25]. Therefore, we hypothesized that VP16 might act downstream of MAVS-Pex through a similar mechanism, interacting with coactivators of IRFs, but not direct on MAVS-Pex. However, until now the signal pathway downstream of MAVS-Pex is still largely unknown, so identification of VP16 target in the MAVS-Pex pathway and revealing the underlying molecular mechanisms require further investigation.

## Conclusions

In summary, our studies define for the first time the contribution of HSV-1 tegument protein VP16 in the evasion of the immediate early antiviral response through MAVS-Pex signaling. This finding leads to a better understanding of the mechanisms of VP16 in dampening the host antiviral signaling and uncovers a new method of HSV-1 invasion against early-stage defense in host cells.

## Methods

### Cells, viruses, antibodies and reagents

HEK293, MEF and HEK293T cells were grown in Dulbecco’s modified minimal essential medium (DMEM) (Gibco-BRL) supplemented with 10% fetal bovine serum (FBS) as described previously [38, 52]. The WT HSV-1 F strain virus was propagated in Vero cells as described previously [38]. Rabbit anti-UL42 polyclonal antibody was made by GL Biochem Ltd. (Shanghai, China). Mouse anti-HA monoclonal antibody (mAb), anti-Flag mAb and anti-β-actin mAb were purchased from Abmart (Shanghai, China). Mouse anti-VP16 mAb was purchased from Santa Cruz Biotechnology (CA, USA). Rabbit anti-viperin polyclonal antibody was purchased from Abcam (Cambridge, MA, USA). BFA was purchased from Selleck (TX, USA). MAVS-KO MEF cells were provided by Dr. Chen Wang.

### Plasmid construction

All enzymes used for cloning procedures were purchased from Vazyme (Nanjing, China). MAVS-Pex-YFP and MAVS-Pex-HA plasmids were subclones from MAVS-Pex-GFP (Addgene, MA, USA). Renilla luciferase plasmid pRL-TK (expressing thymidine kinase [TK]) was purchased from Promega (Madison, WI, USA). Oligonucleotides encoding shRNA-VP16 was synthesized and inserted into the pSuper.retro.puro vector (Oligoengine, LA, USA). Viperin reporter plasmids Viperin-Luc and Vig1-Luc were gifts from Dr. Katherine A. Fitzgerald. Plasmid PTS1-DsRed was a gift from Dr. Jonathan C. Kagan.

### Transfection, BFA treatment and DLR assay

MEF cells were transfected with Lipofectamine® LTX (Invitrogen, CA, USA) according to the manufacturers’ recommendations. HEK293 cells were preincubated with 50 ng/ml BFA and infected with or without WT HSV-1 in the presence of BFA. At 2 h post infection, the supernatants were discarded and replaced by cell culture containing 50 ng/ml BFA, and cells were cotransfected with viperin luciferase reporter plasmid Viperin-Luc and internal control pRL-TK, with or without indicated plasmids in the presence of BFA, by standard calcium phosphate precipitation [53]. Luciferase assays were performed with a dual-specific luciferase assay kit (Promega) as previously described [38, 44].

### RNA isolation, qRT-PCR

Total RNA was extracted from HEK293 cells with TRIzol (Invitrogen, CA, USA) according to the manufacturer’s manual. Samples were digested with DNase I and subjected to reverse transcription. The cDNA was used as a template for qRT-PCR to detect the accumulation of indicated mRNA as previously described [44]. The primers used for RT-PCR analysis are as follows: Viperin tggtgaggttctgcaaagtag (forward) and tcacaggagatagcgagaatgtc (reverse), GAPDH tgacctcaactacatggtttacatgt (forward) and agggatctcgctcctggaa (reverse).

### Western blot analysis

WB analysis was performed as previously described [38, 52].

### Statistical analysis

Data are represented as mean ± SD when indicated, and Student’s *T*-test was used for all statistical analyses with the GraphPad Prism 5.0 software. Differences between groups were considered significant when *P*-value was <0.05.

## Abbreviations

BFA: Brefeldin A
DLR: Dual-luciferase reporter
HSV-1: Herpes simplex virus 1
ISG: IFN-stimulated gene
MAVS: Mitochondrial antiviral signaling protein
MAVS-Pex: Peroxisomal MAVS
MOI: Multiplicity of infection
